# Case Report: Neonatal Varicella Acquired From Maternal Zoster

**DOI:** 10.3389/fped.2021.649775

**Published:** 2021-03-03

**Authors:** Jeffrey W. Lai, Timothy Ford, Sarah Cherian, Anita J. Campbell, Christopher C. Blyth

**Affiliations:** ^1^School of Medicine, The University of Western Australia, Crawley, WA, Australia; ^2^Department of Infectious Diseases, Perth Children's Hospital, Nedlands, WA, Australia; ^3^Wesfarmers Centre of Vaccines and Infectious Diseases, Telethon Kids Institute, Nedlands, WA, Australia

**Keywords:** neonatal varicella, maternal zoster, maternal immunosuppression, trans-placental immunity, varicella zoster immune globulin

## Abstract

The incidence of neonatal varicella has decreased dramatically since the introduction of the varicella vaccination. Although the varicella zoster virus is often associated with a mild infection, it may cause severe morbidity and mortality, particularly in the neonatal period and immunocompromised hosts. We report a case of neonatal varicella acquired from maternal zoster in a mother on biological immunosuppressive therapy. Following the diagnosis, the baby improved on antiviral therapy without any neurological sequelae. This case highlights the limited published data on neonatal varicella following herpes zoster reactivation to inform practice. This includes the role of varicella zoster immunoglobulin in neonates exposed to maternal zoster, the degree of trans-placental immunity and optimum antiviral dosing and duration.

## Introduction

The varicella zoster virus (VZV) is a member of the Herpesviridae family and is responsible for causing primary varicella infection (chickenpox) and herpes zoster (shingles). Neonatal varicella is most commonly acquired by VZV transmission from a mother with active primary varicella infection to her baby either during the intrauterine period in the last three weeks of pregnancy or postnatally (1). Although VZV infection is often associated with mild disease, it may cause disseminated infection that is associated with increased mortality particularly in neonates (2). The severity of the illness is dependent on the transfer of specific protective antibodies from mother to baby *in utero* (1). Therefore, the clinical sequelae of a neonatal exposure to maternal zoster is thought to be milder due to protective maternal VZV-specific IgG compared to maternal primary varicella (1).

We present a clinical case of varicella in a neonate whose mother was on infliximab therapy and developed maternal herpes zoster day three post-delivery.

## Case Presentation

**Figure 1:**
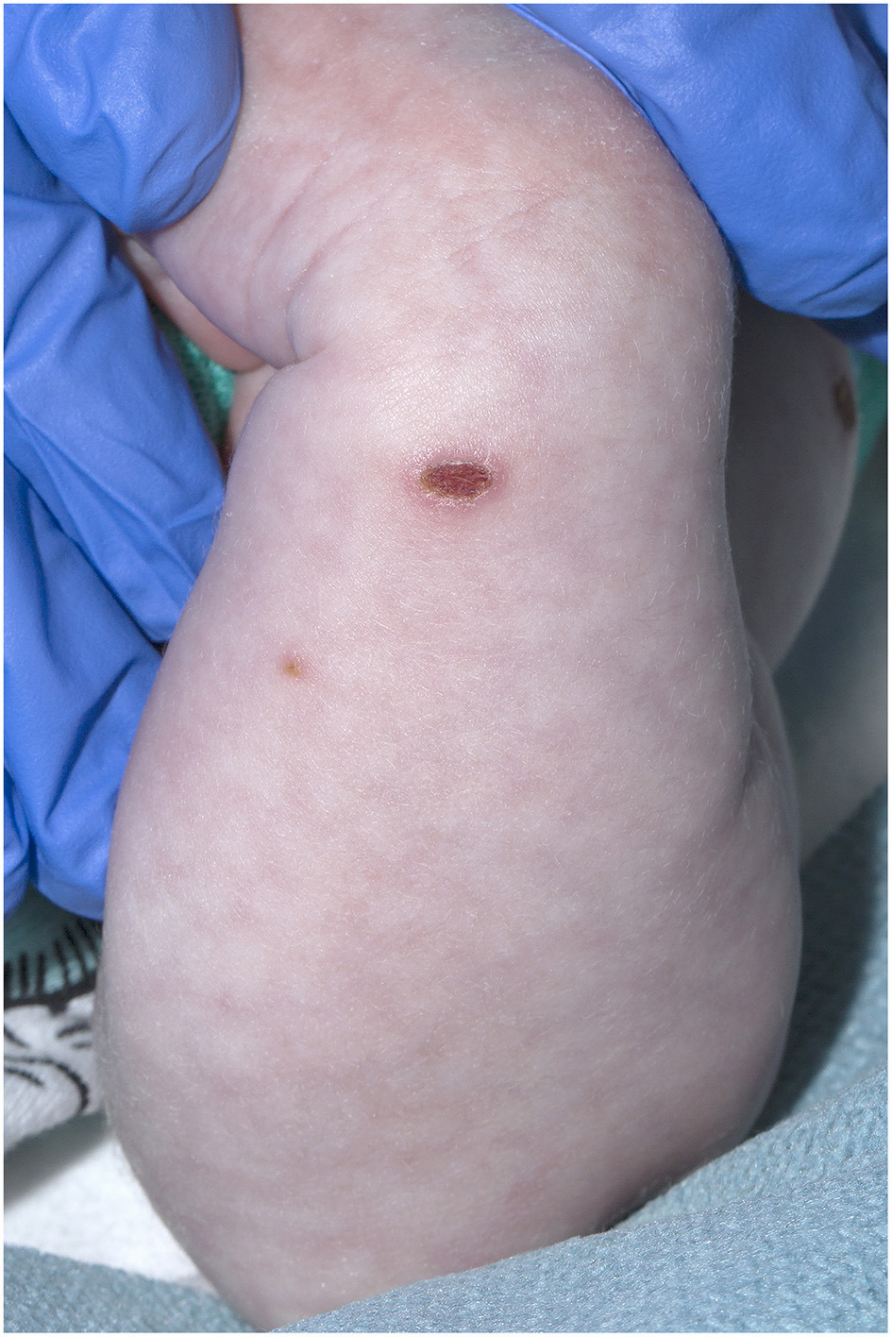
Crusted varicella lesions in a 21-day old child - Arm.

**Figure 2:**
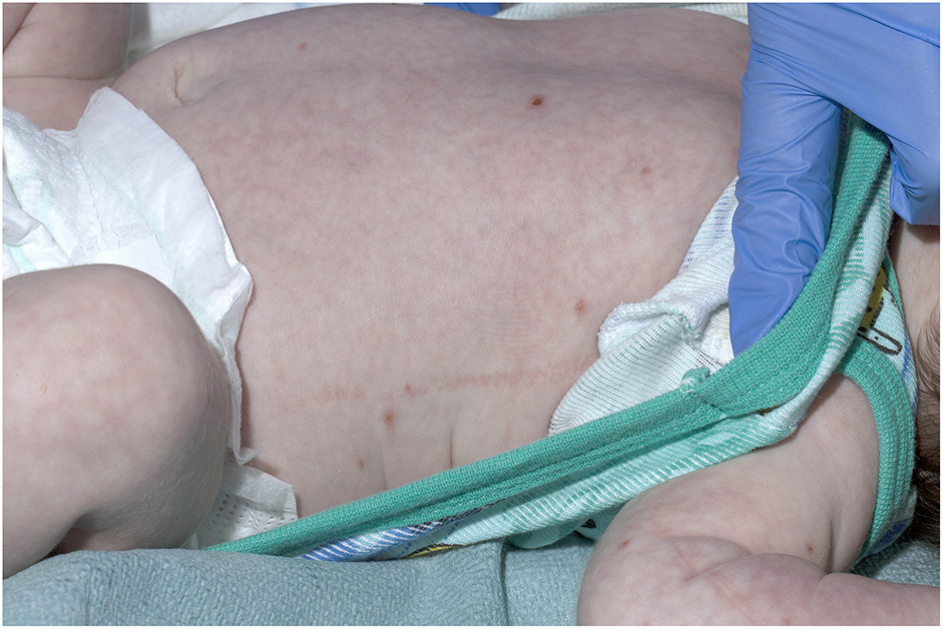
Crusted varicella lesions in a 21-day old child - Trunk.

A 21-day old male (39^+2^ weeks gestation) born via elective cesarean section, was referred to a tertiary pediatric hospital from a private pediatrician with a vesicular rash that had been present for five days. On presentation, the neonate had a history of reduced feeding, was afebrile, and had oxygen saturations of 98% in room air. There were between 15 and 20 vesicular lesions over the trunk, arms, legs, face and scalp with new onset cutis marmorata (Figures 1, 2). No other abnormality was noted on examination. Skin lesions, blood and cerebrospinal fluid (CSF) were sampled, herpes simplex virus type 1 and 2 and VZV polymerase chain reaction (PCR) tests performed, and the patient was commenced on intravenous (IV) acyclovir (20 mg/kg 8 h) on day one of admission. Full blood count demonstrated mild monocytosis (1.94 × 10^9^/L) and thrombophilia (467 × 10^9^/L) with normal liver and renal function tests.

The mother provided a history consistent with herpes zoster on her breast and upper chest (T3 dermatome), which was noted at day three post-delivery. VZV infection was confirmed via PCR and contemporaneous maternal varicella serology demonstrated IgG positivity. Her General Practitioner (GP) prescribed valacyclovir and breast feeding from the affected side was temporarily ceased, although the neonate continued to receive expressed breast milk. Varicella zoster immunoglobulin (VZIG) was not offered to the neonate following his mother's zoster eruption. This was the mother's first episode of zoster; she had primary varicella infection when she was five years old. The mother has a seven-year history of Crohn's disease managed with infliximab and mercaptopurine. The last dose of infliximab was four months prior to delivery.

Skin lesion viral swabs, blood and CSF from the neonate returned positive for VZV deoxyribonucleic acid (DNA). The CSF sample was clotted and a cell count was subsequently not performed. VZV-specific IgG and IgM were negative in the neonate. An equivocal maternal VZV-specific IgG level was identified (using the DiaSorin Liaison XL) retrospectively on stored maternal serum taken during the first trimester of the pregnancy.

As the possibility of varicella meningitis could not be excluded based on the CSF result, the neonate was treated with IV acyclovir (20 mg/kg 8 h) for 10 days. He improved, with no new lesions occurring following commencement of therapy. The patient was discharged on day three to complete intravenous acyclovir therapy via the Hospital in the Home service. Ongoing follow up has been arranged with audiology and general pediatrics to assess for any long-term neurological sequelae.

## Discussion

The incidence of neonatal varicella has dramatically decreased in the post vaccination era, with the varicella vaccine first included in the Australian National Immunization Program in 2005 (3). The Australian Pedatric Surveillance Unit reported that between 2006 and 2009, the incidence of neonatal varicella was 0.7 per 100 000 live births per year – an 85% reduction compared to the pre-vaccination period between 1995 and 1997 (4). There are limited contemporary data available to guide prevention and management of neonatal varicella. Indirect evidence from clinical trials showed that treating varicella in a small number of immunocompromised children in the 1980s with acyclovir lowered the risk of severe complications including pneumonitis (5). Recommendations to help clinicians identify at-risk neonates are lacking along with guidance about when to initiate acyclovir therapy, optimum acyclovir dosing and treatment duration.

The incubation period of VZV from inoculation until infection is usually 14–16 days (1). The time between when the rash was first noticed in the mother until the onset of rash in the neonate was 13 days, falling within the incubation period of VZV. It was considered that direct exposure to lesions of the breast was the most likely mode of acquisition. Although the neonate received express milk while the mother had herpes zoster reactivation, this may not have been the source of the infection as breast milk has not been reported to be a significant route of transmission for VZV (6). The source of the virus that infected the neonate may also have originated from the mother's saliva from actions such as kissing (7).

In this case, the mother had an equivocal VZV-specific IgG serology antenatally. This result suggests she did have a degree of varicella immunity, consistent with previous varicella infection and positive serology at the time of illness. The equivocal result may have been due to sensitivity of serological testing, which may not be able to detect low-positive VZV titers (8). Despite this, there was no significantly detectable transfer of protective VZV-specific antibodies to the neonate. This suggests that there were inadequate levels of protective maternal antibodies and therefore minimal *in utero* transfer resulting in inadequate protection of the neonate.

The mother had Crohn's disease and was receiving infliximab therapy during her pregnancy, both of which have been reported to increase the risk of herpes zoster viral reactivation as infliximab impairs the ability to mount an immune response (9–11). The varicella vaccine is recommended to VZV-IgG negative women who are planning pregnancy (3). However, only live-attenuated varicella vaccines are available in Australia and are thus contraindicated in individuals undergoing immunosuppressive therapy (1). This case highlights the importance of optimizing immunizations in individuals prior to commencing immunosuppressive therapy, as they are at increased risk of developing herpes zoster, where possible and the role of alternative zoster vaccines (e.g., Shingrix, GlaxoSmithKline Biologicals SA) in the immunosuppressed host setting.

Biological immunosuppressive agents such as infliximab can cross the placenta and enter the fetal circulation (12). This in combination with an underdeveloped immune system in neonates raises the question whether VZIG or oral acyclovir has a role to play in preventing disease regardless of maternal VZV-IgG status or disease phenotype in the setting of significant maternal immunosuppression (13). Most consensus guidelines, including the American Academy of Pediatrics Red Book® state that VZIG is only indicated if the mother develops a primary varicella infection five days before delivery or within 48 hours after delivery (1). VZIG is not indicated if the mother has zoster or detectable varicella IgG as this suggests evidence of maternal immunity to VZV and an ability to provide trans-placental immunity (1). This was not evident in our case. The current understanding of the effects of exposure to biological immunosuppressive antibodies in gestation on the neonatal immune system is limited. Further studies are required to determine whether VZIG or oral acyclovir prophylaxis is effective in preventing varicella infection in neonates exposed to immunosuppressive monoclonal antibodies.

Neonates have an immature immune system, particularly their adaptive immunity. Their innate immune system, which has no memory or specificity, therefore plays a major role in the control of neonatal varicella (1). Due to these factors, the patient is recommended to receive the varicella vaccine scheduled at 18 months on the Australian national immunization program, despite evidence of primary varicella infection, to develop a strong adaptive immune response to the virus (1, 3). The rotavirus vaccine at six weeks of age was also recommended to the patient despite some guidelines recommending against administering live attenuated vaccines to infants before the age of six months if exposed to monoclonal antibodies *in utero* (14). In support of this, a recent study demonstrated that the rotavirus vaccine was safe in infants who have been exposed to biologic immunosuppressive therapy during gestation (14).

This case of neonatal varicella developed in the setting of maternal immunosuppression with clinical and virological evidence of maternal zoster infection, despite a maternal history of primary varicella infection. Care must be taken with immunosuppressed mothers who develop zoster as the degree of effective trans-placental immunity is uncertain. Whether VZIG should be administered to neonates born to mothers on significant immunosuppressive therapy and exposed to zoster requires further consideration and research, particularly in a climate of increasing use of biologic immunosuppressive therapies. Furthermore, contemporary data regarding the management of neonatal varicella is limited and ongoing national surveillance (4) and research is required to guide best practice.

## Data Availability Statement

The original contributions presented in the study are included in the article/supplementary material, further inquiries can be directed to the corresponding author/s.

## Author Contributions

TF, SC, AC, and CB were directly involved with the patient's care and contributed to the preparation of the manuscript. JL wrote the manuscript with input and comments from all authors. All authors read and approved the final manuscript.

## Conflict of Interest

The authors declare that the research was conducted in the absence of any commercial or financial relationships that could be construed as a potential conflict of interest.
